# Mechanistic Study of Atomic Oxygen Erosion on Polyimide Under Electric Fields: A Molecular Dynamics and Density Functional Theory Approach

**DOI:** 10.3390/molecules29225353

**Published:** 2024-11-14

**Authors:** Shengrui Zhou, Li Zhang, Liang Zou, Bilal Iqbal Ayubi, Yiwei Wang

**Affiliations:** School of Electrical Engineering, Shandong University, Jinan 250061, China

**Keywords:** polyimide, atomic oxygen, electric field, molecular dynamics, density functional theory

## Abstract

Polyimide (PI) is widely used in aerospace applications due to its superior insulating properties. However, the high concentration of atomic oxygen (AO) in low Earth orbit leads to significant performance degradation in PI, and the underlying mechanism of AO erosion under an electric field remains unclear. This study utilizes molecular dynamics simulations to model AO erosion on PI under various electric field strengths and explores the corresponding degradation mechanisms. The results indicate that the presence of an electric field exacerbates the degradation of PI by AO. AO erosion elevates the polymer’s temperature, and the combined effects of thermal and electric stresses increase the polymer’s free volume, loosening its structure and accelerating degradation. The quantity of AO-induced erosion products increases with rising electric field strength, causing more large carbon chains to detach from the polymer surface. Density functional theory (DFT) calculations further reveal that the electric field reduces the frontier orbital energy gap in PI molecules, making AO erosion reactions more thermodynamically favorable. This work provides an atomic-level insight into the degradation mechanism of PI under AO erosion in electric fields and offers a theoretical basis for future studies on polymer resistance to AO erosion in space environments.

## 1. Introduction

Polyimide (PI) is widely recognized for its excellent insulating properties, mechanical strength, and thermal stability, making it an essential material for flexible substrates in solar arrays, flexible optical solar reflectors, and electrical insulation in aerospace applications [1,2,3,4,5,6]. Typically, functional PI films have a low dielectric constant of between approximately 3.0 and 3.5 and can withstand temperatures up to around 352 °C [7,8]. However, the space environment, particularly in low Earth orbit (LEO), exposes polymers like PI to various detrimental factors, including atomic oxygen (AO) erosion, charged particles, significant temperature gradients, intense ultraviolet radiation, and micrometeoroid impacts, all of which contribute to both electrical and mechanical degradation [9,10,11]. Such degradation can result in critical issues, including discharge breakdown, flashover, and electrostatic discharge, significantly shortening the operational life of spacecraft.

Due to the high velocity of orbital spacecraft (approximately 7.8 km/s), AO impacts surfaces with an effective kinetic energy of 4–5 eV, making it a primary threat to polymers used in LEO [12,13]. Studies by Hu et al. revealed substantial mass loss in PI exposed to AO, with the surface developing a “carpet-like” morphology. This is attributed to high-energy AO reacting with the PI surface, causing chemical bond dissociation and the formation of volatile compounds [14]. Ground-based experiments conducted by Qi et al. showed that after 5 h of exposure to an AO environment, PI exhibited visible wear, and with continued exposure, color fading, thickness reduction, and cracking became more apparent [15]. Li et al. investigated the deterioration of Kapton PI under AO erosion and found that the surface rapidly transitioned from smooth to rough, with numerous micro-holes and cracks forming [16]. Zhang’s experiments demonstrated that AO erosion leads to the breakdown of chemical bonds, such as C-H and C-O, destroying the carbon-hydrogen backbone and significantly degrading the material’s mechanical properties and surface morphology [17]. Jeon’s comparative analysis of AO and nitrogen molecule bombardment revealed that AO-induced oxidation and desorption are the primary degradation mechanisms. AO’s unpaired electrons react with carbon atoms on the PI surface, breaking C-C and C-H bonds [18].

With the advancement of high-power, high-voltage space technologies, the stability of PI in extreme space conditions has garnered increasing attention [19,20]. Studies by Shang et al. indicate that varying operational voltages in the space environment influence the internal electric field distribution within PI. The applied voltage, combined with space-deposited charges, forms an electric field. When the electric field strength exceeds the breakdown threshold of PI, dielectric discharge occurs, damaging the material’s electrical properties and structural integrity [21]. Research by Diaw et al. on PI used in space under different electric fields and temperatures showed that at low electric field strengths and temperatures, PI maintains good insulation properties. However, as both the electric field strength and temperature increase, PI’s conductivity rises, significantly reducing its insulation capacity and eventually leading to dielectric breakdown [22]. Wang et al. investigated the decomposition of PI composites under high temperatures and strong electric fields, revealing that under intense electric fields, electric field stress induces tensile forces on PI materials. When the electric field strength and temperature exceed certain thresholds, C-N and C=O bonds break, leading to a decline in insulation properties and ultimately resulting in decomposition and dielectric breakdown [23].

Ground-based AO exposure experiments cannot fully elucidate the molecular-level interactions and structural changes occurring in polymers during AO erosion [24,25,26]. Molecular dynamics (MD) simulations based on the reactive force field (ReaxFF) methodology enable the detailed study of AO erosion processes on polymers, analyzing changes in structure, energy, and various physicochemical properties over very short time scales. This method has been widely adopted in studies of space polymer materials [27]. Using MD simulations, Wei et al. analyzed the damage mechanisms and mechanical degradation of Kapton and Upilex-S PI materials under AO erosion. Their results showed that AO erosion rapidly raises the surface temperature of the materials, particularly at higher incident angles, making them more prone to severe damage [28]. Qiao et al. used reactive MD simulations to study the degradation mechanisms of PI materials under AO bombardment, finding that AO gradually penetrates from the surface into the material through chemical reactions, and the erosion rate increases with rising temperature, leading to a significant increase in reaction products [29]. Lin et al. combined MD simulations with experimental studies to investigate the structural and performance changes in PI films under high-temperature thermal aging. Their research showed that the thermal decomposition of PI primarily generates gases such as CO_2_, CO, and NH_3_. Although PI materials with different structures produce similar decomposition products, their thermal stability differs [30]. These findings provide a foundation for understanding the molecular-scale processes that lead to degradation in PI films and guide the development of materials with improved resistance to AO erosion and enhanced performance stability in space environments.

Molecular dynamics simulations, however, struggle to capture electronic structure changes and molecular reactivity under various conditions. In contrast, density functional theory (DFT) calculations provide precise insights into the molecular orbitals and electronic properties of different molecular states [30,31,32,33]. Gong et al. utilized DFT to explore the reaction mechanism of PI under AO erosion, revealing that AO erosion is an electrophilic reaction, preferentially targeting regions with high electron density in PI molecules, which served as reactive sites [34]. Li et al. employed DFT to predict the thermal decomposition temperature and electronic structure of PI films with different structures, optimizing molecular design by adjusting the main and side chain structures to enhance thermal performance [35]. Zheng et al. studied the influence of frontier orbital distribution on the dielectric constant through DFT calculations, finding that the introduction of tert-butyl structures increased the free volume between PI molecular chains, reducing intermolecular interactions and lowering the dielectric constant [36].

This study combines ReaxFF-based reactive molecular dynamics simulations with DFT calculations to systematically investigate the degradation behavior of PI under AO erosion at different electric field strengths. By analyzing normalized mass and temperature changes during AO erosion, identifying the quantity and reaction pathways of small molecular products, and studying the variations in free volume and frontier orbital properties under different electric fields, this work elucidates the degradation mechanism of PI under AO erosion. It provides theoretical insights into the mechanism of PI degradation under AO in electric fields, offering valuable guidance for the design and optimization of polymer materials resistant to AO erosion in space environments.

## 2. Results and Discussion

### 2.1. Polyimide Erosion Kinetics Under Electric Fields

**Figure 1:**
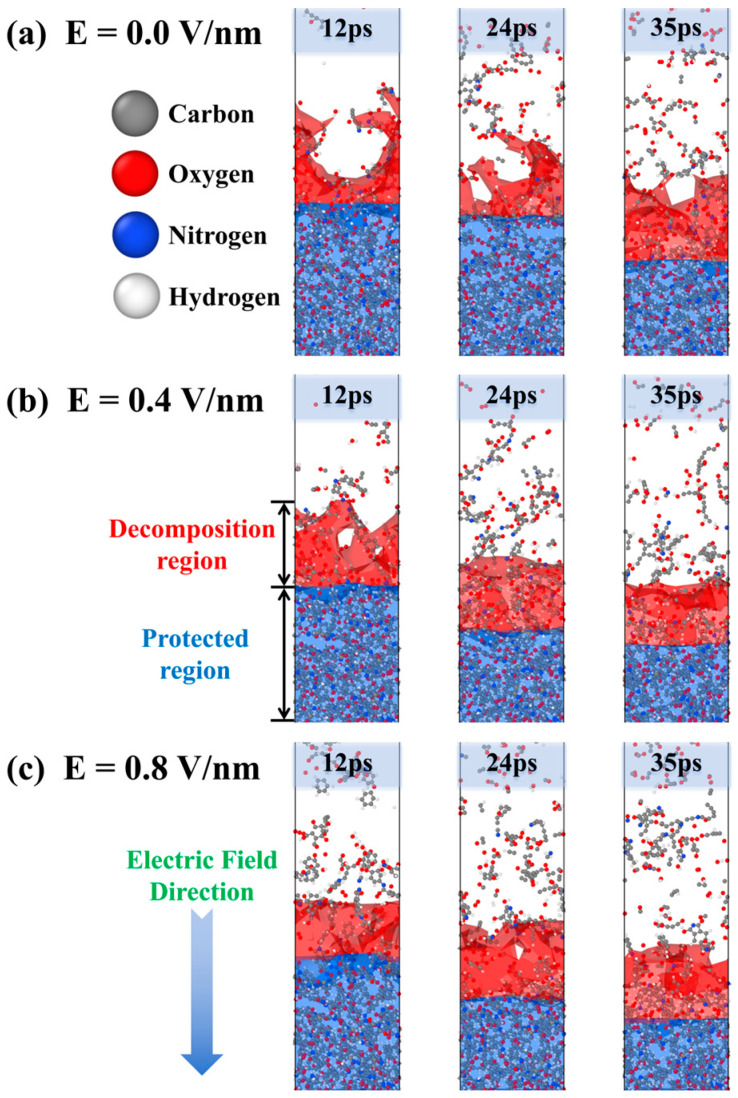
Snapshots of the model erosion during AO exposure under electric field strengths of (**a**) E = 0.0 V/nm, (**b**) E = 0.4 V/nm, and (**c**) E = 0.8 V/nm.

Figure 1 shows the structural evolution of the PI model during AO erosion under an electric field. The AO-eroded region is termed the decomposition region, while the area below, protected by small molecular fragments and intact polyimide, is referred to as the protected region. As shown in Figure 1a, continuous AO erosion gradually decomposes the model into numerous small molecular products that float above the polymer. As the reaction proceeds, the molecular structure within the decomposition region becomes increasingly loose and cracks, extending the erosion range. The interface between the decomposition and protected regions shifts downward, and molecular motion accelerates, raising the model’s temperature. Under a 0.4 V/nm electric field, as shown in Figure 1b, the decomposition rate of the model accelerates significantly, with a marked increase in small molecular byproducts. In both the no-electric-field model and the 0.4 V/nm electric field model, the decomposition byproducts mainly consist of small molecules and short carbon chains, with AO erosion dominating. When the electric field strength is increased to 0.8 V/nm, as shown in Figure 1c, the primary products become long carbon chains, indicating that the electric field and high temperature are now the dominant factors in PI degradation, with AO primarily colliding with free carbon chains.

**Figure 2:**
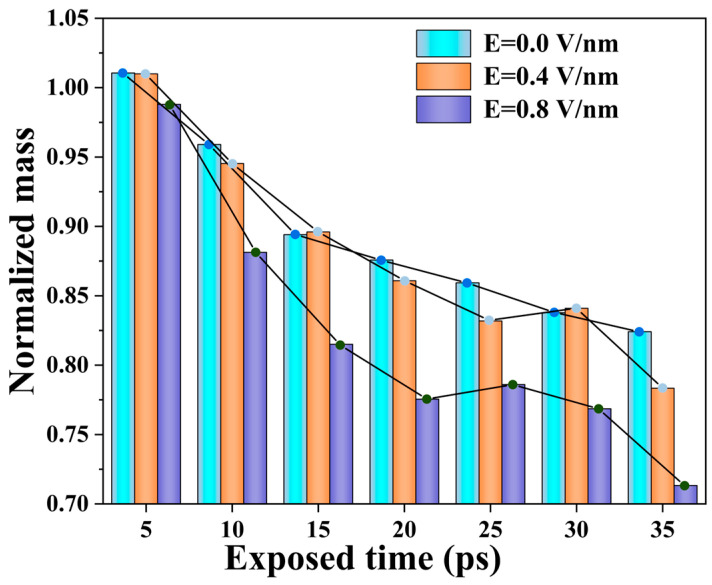
Normalized mass changes during AO erosion under different electric field strengths.

To quantitatively assess the extent of PI degradation during AO erosion under different electric field strengths, the normalized mass change of the model during AO erosion was calculated. The normalized mass is obtained by dividing the remaining mass of the model by its initial mass, as shown in Figure 2. In the first 15 ps, the normalized mass changes of PI models under no electric field and in a 0.4 V/nm electric field are nearly identical. As AO continues to inject energy into the model and reaches the PI decomposition threshold, the PI molecules begin to degrade extensively, leading to a significant decrease in mass. Under a 0.8 V/nm electric field, the polymer maintains a relatively fast degradation rate from the start of the simulation until the reaction ends. The strong electric field causes large-scale PI decomposition early in the simulation, and the degradation rate does not accelerate further with the introduction of AO. Instead, AO primarily collides with the degradation products. Between 25–35 ps at E = 0.4 V/nm and 20–25 ps at E = 0.8 V/nm, the normalized mass shows an increase, due to some decomposition products returning to the polymer surface after colliding with AO, offering some protection and slowing further degradation of the underlying polymer.

### 2.2. Free Volume and Temperature Evolution During AO Erosion

**Figure 3:**
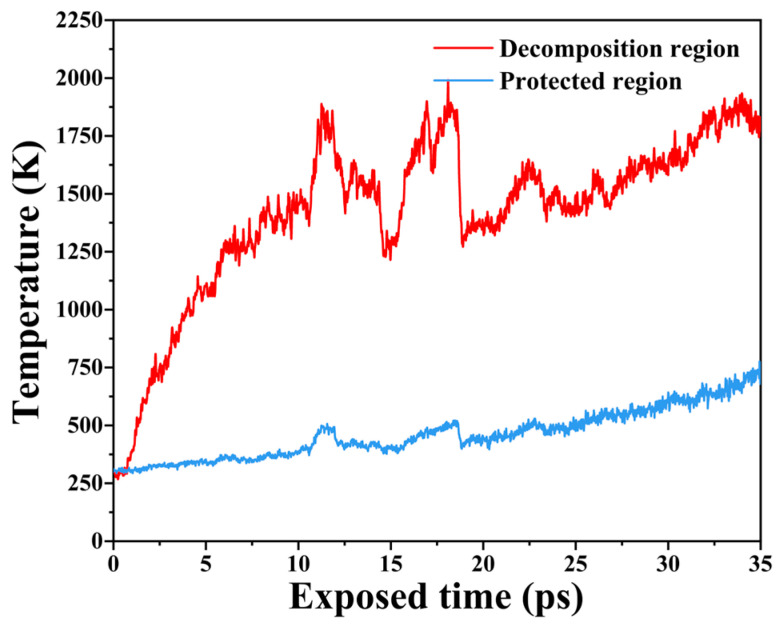
Temperature variations in the decomposition and protected regions during AO erosion.

To explore the temperature evolution of the PI model during AO erosion, temperature changes in both the decomposition and protected regions were calculated, as shown in Figure 3. The temperature of the decomposition region continues to rise due to AO collisions and oxidation reactions with PI molecules, peaking at approximately 1800 K at 12.5 ps before decreasing. The surface PI molecules crack into small molecules, which escape and carry away heat, causing the temperature to drop to between 12 and 15 ps. At this point, the interface between the decomposition and protected regions descends, exposing PI molecules in the protected region to AO, continuing the reaction and causing the temperature to rise again. Free small molecules and PI molecules from the decomposition region provide protection for the underlying polymer. The temperature in the protected region rises more slowly, mainly due to heat exchange with the decomposition region.

**Figure 4:**
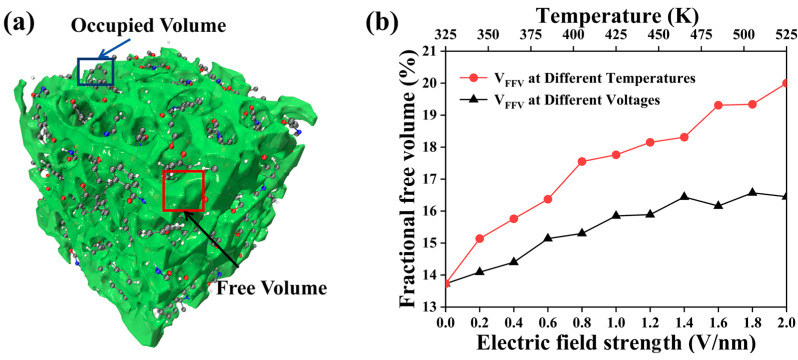
(**a**) Free volume distribution in the PI model and (**b**) changes in free volume under different temperatures and electric field strengths.

Free volume refers to the voids or non-densely packed regions within a polymer. These voids influence molecular chain movement, material diffusion properties, and the rate of AO penetration into the polymer structure [37,38]. To better analyze the void distribution in the amorphous region of PI under different voltages and temperature conditions, a spherical probe with a radius of 0.1 nm was used to move along the van der Waals surface to generate a Connolly surface. The volume between the probe atom and the van der Waals surface is considered free volume [39]. Figure 4 shows the changes in free volume under different electric field strengths and temperatures. Green areas represent free volume regions, while the remaining areas represent occupied atomic volume. The fractional free volume (FFV) is used to describe the relative changes in free volume under different conditions, as shown in Figure 4b. The expression for FFV is

(1) $$V_{FFV}=\frac{V_{f}}{V_{f}+V_{0}}\times 100\%$$

where V_f_ is the free volume, V_0_ is the occupied volume, and V_FFV_ is the fractional free volume distribution.

As the temperature and electric field strength increase, the free volume of PI also increases. With rising temperatures, the thermal motion of polymer molecular chains intensifies, widening the gaps between molecular chains and increasing free volume from 17.3% at 300 K to 20.1% at 525 K, making the structure looser [40]. Under the electric field, polarized molecules reorient and rearrange along the field direction, inducing local space changes and connecting isolated free regions, increasing free volume. At an electric field strength of 2.0 V/nm, the free volume rises to 16.5%. Both temperature and electric field disrupt weak interactions between PI molecules, changing the molecular morphology and further increasing free volume.

### 2.3. AO Erosion Product Analysis

**Figure 5:**
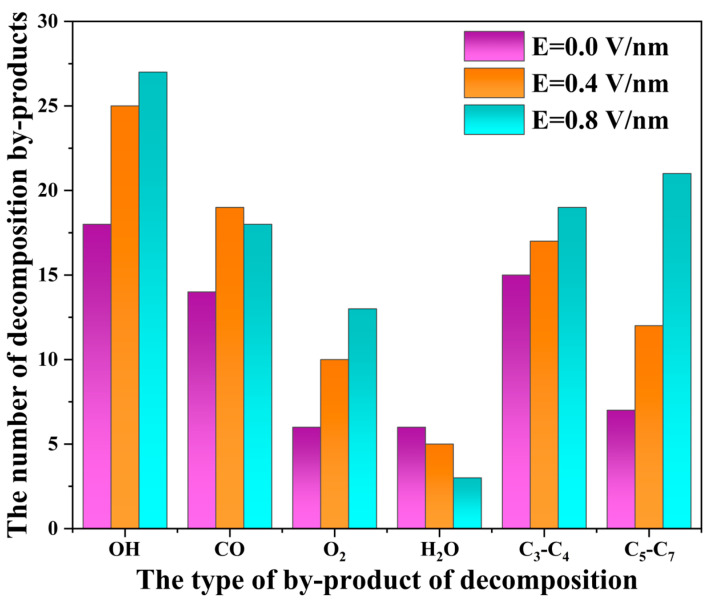
Variations in the types and quantities of products during AO erosion simulation.

To analyze the products generated during AO erosion under different electric field strengths, the main products extracted after 35 ps of simulation were quantified, as shown in Figure 5. The primary products collected were OH, CO, O_2_, and H_2_O. Among all AO erosion processes, OH was the most abundant product, due in part to the high abundance of hydrogen atoms in PI, facilitating frequent hydrogen abstraction reactions by AO. The electric field also alters the electron distribution in PI molecules, changing the degradation pathways and making bond polarization and cleavage more likely, allowing AO to more easily extract hydrogen atoms from PI molecules. The charge redistribution of each atom in the ReaxFF force field under the electric field makes OH more susceptible to escaping from the polymer surface, reducing its subsequent reactivity. As a result, the amount of H_2_O produced decreases with increasing electric field strength. Additionally, the electric field redistributes the electron density in PI molecules, making oxygen atoms on the imide ring more likely to act as electron donors. The increased local electron density intensifies surface oxidation, leading to an increase in O_2_ production with stronger electric fields. Without an electric field or under weak fields, AO erosion primarily drives PI degradation, with AO preferentially attacking weak or easily oxidized bonds in PI chains, breaking them into shorter carbon chains (e.g., C_3_–C_5_). Under strong electric fields, direct forces on the bonds polarize and break them, forming longer carbon chains (e.g., C_5_–C_7_), with both AO erosion and PI degradation occurring simultaneously.

**Figure 6:**
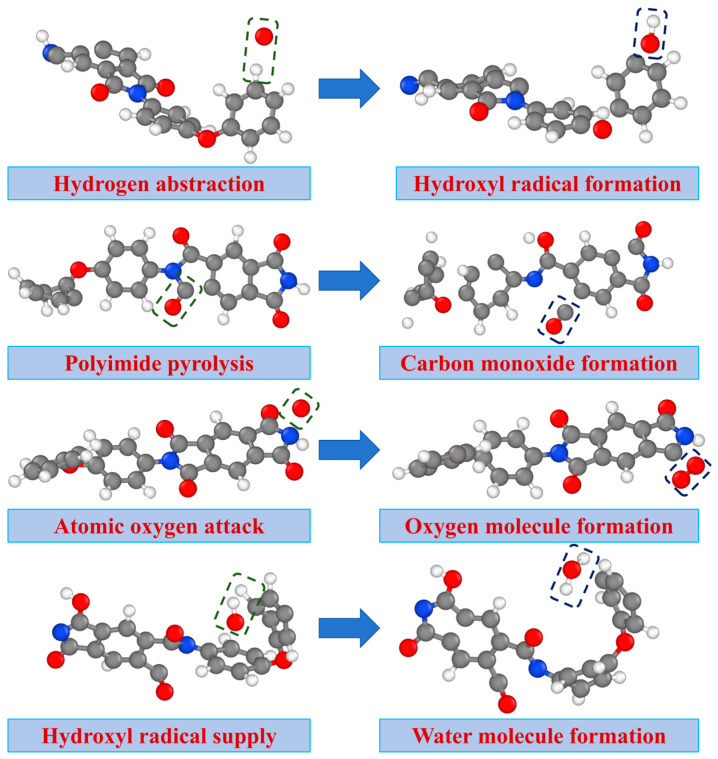
Snapshots of the main products generated during AO erosion.

Figure 6 shows snapshots of the main products generated during the AO erosion reaction. AO’s unpaired electrons and high electronegativity make it prone to abstracting electrons from C-H bonds, forming hydroxyl radicals with hydrogen atoms. These radicals subsequently react with additional hydrogen atoms to produce water. As the erosion continues, the temperature of the polymer model rises, and when it reaches the thermal decomposition threshold of PI, C-N and C-C bonds in the imide ring break, generating CO. High temperatures provide enough energy to overcome bond energies in the imide ring, causing the molecular structure to collapse. AO oxidation reactions with the benzene ring generate OH and CO, which become the most abundant degradation products. The synergy between PI thermal decomposition and AO oxidation provides heat to drive the reaction, while intermediates from thermal decomposition offer additional reaction sites for AO. High temperatures and electric fields together activate the electronic distribution within PI molecules, lowering bond energy barriers and accelerating AO attacks on C=O bonds in the imide ring, inducing electron transfer and generating O_2_.

**Figure 7:**
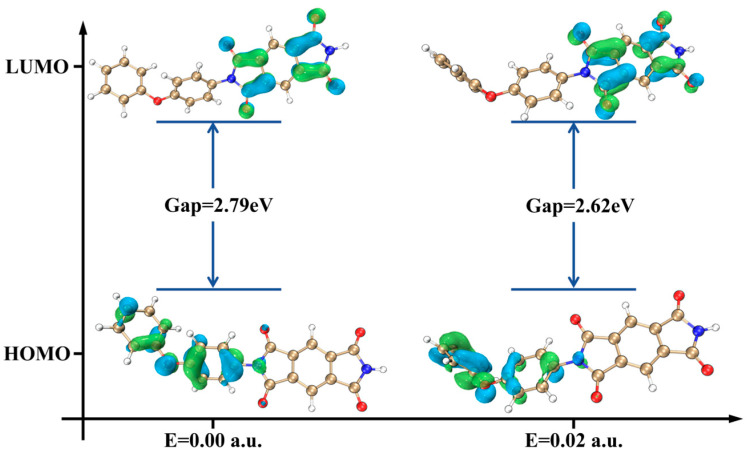
Changes in frontier orbitals and energy gap of PI molecules under electric field influence.

To explore the influence of electric fields on the electronic structure and reactivity of PI molecules, the energy gap between the highest occupied molecular orbital (HOMO) and the lowest unoccupied molecular orbital (LUMO) was calculated, as shown in Figure 7. The energy gap decreases from 2.79 eV to 2.62 eV under the electric field, indicating that PI molecules are more likely to undergo electron transfer, making it easier for AO to extract electrons, enhancing the reactivity of PI with AO and accelerating the material’s oxidation and degradation. The electric field also induces electron redistribution, rearranging PI’s electronic structure, reducing its stability, and accelerating both thermal decomposition and the AO reaction, further hastening AO erosion.

## 3. Methods and Models

The AO erosion simulations were performed using the Reax module in the Large-scale Atomic/Molecular Massively Parallel Simulator (LAMMPS) [41]. The modeling process was carried out in BIOVIA Materials Studio (MS), with relaxation employing the COMPASS III [42] force field and a time step of 1 fs. The force field parameters for AO erosion simulations were sourced from Rahmani et al. [43]. DFT calculations were conducted to reveal frontier orbital information of PI under electric fields, with all DFT calculations performed using the Gaussian 16 [44] software package. Structure optimization and single-point energy calculations were conducted using the B3LYP/6-31G(d) basis set.

**Figure 8:**
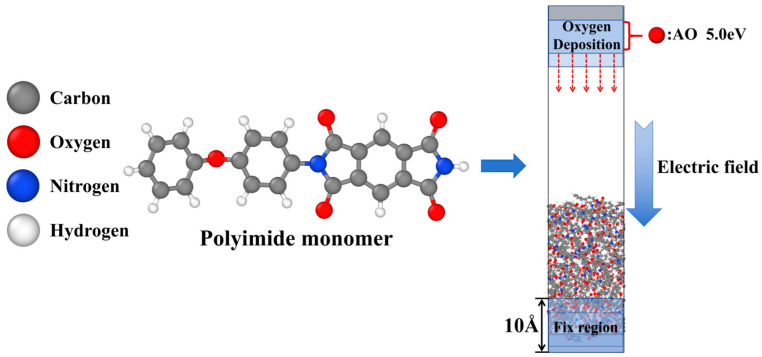
The PI monomer structure and the relaxed equilibrium cell structure.

Figure 8 presents the PI monomer and relaxed model structure used in this study. First, 50 PI monomers were placed into a periodic cell (24 Å × 24 Å × 42 Å) for NPT ensemble relaxation over 500 ps at 298 K and 1 atm pressure. The model’s density converged to equilibrium (ρ_PI_ = 1.35 g/cm^3^), near the standard density of 1.41 ± 0.2 g/cm^3^. The relaxed model size was 24.02 Å × 24.02 Å × 41.53 Å. The periodic boundary in the Z direction was removed, extending the model to 120 Å, followed by an NVT ensemble relaxation over 500 ps at 300 K. To prevent model displacement due to AO impacts, the bottom 10 Å of atoms were fixed as an impact buffer layer.

Based on the space environment [45,46,47], AO with kinetic energy of 5 eV was introduced 50 Å above the model surface at a rate of 5 atoms/ps, with a time step of 0.1 fs and a total simulation time of 35 ps. Products detached from the model surface were recorded every 1 ps to track AO erosion reaction products. Two electric fields, 0.4 V/nm and 0.8 V/nm, were applied along the negative Z axis to explore the degradation process. All AO erosion simulations were conducted under the NVE ensemble to observe surface reactions and structural changes in real time.

## 4. Conclusions

This study systematically investigated the degradation behavior of PI under AO erosion in different electric field environments using reactive molecular dynamics simulations. The degradation mechanisms, mass changes, temperature evolution, and free volume changes during AO erosion were analyzed, along with the types and quantities of products detached from the model surface. DFT calculations revealed how electric fields affect frontier orbitals in PI molecules. The following conclusions were drawn:

During AO erosion, PI molecular chains degrade gradually, with small molecules detaching from the polymer surface. The interface between the degraded and unaffected regions moves inward over time. Under a 0.4 V/nm electric field, AO erosion accelerates, and PI degradation rates increase due to the combined effects of electric field and temperature. At 0.8 V/nm, degradation rates stabilize, with thermal-electric synergy becoming the dominant degradation factor.

AO erosion causes rapid temperature increases in the decomposition region. As small molecules escape from the polymer surface, they carry away heat, leading to a temperature drop. Heat exchange between the decomposition and protected regions raises the temperature in the protected area. Increasing electric field and temperature expand PI’s free volume, loosening the structure and exacerbating AO erosion.

AO erosion generates a large number of small molecular products, with OH radicals being the most abundant due to the hydrogen-rich nature of PI. As electric field strength rises, the total degradation product yield increases, with more large carbon chains forming. The reduction in frontier orbital energy gaps makes AO erosion reactions more likely.

This study provides atomic-level insights into the degradation mechanisms of PI under AO erosion in space environments, offering a theoretical foundation for designing and optimizing space polymer materials to resist AO erosion under electric fields.

## Data Availability

The data presented in this study are available on request from the corresponding author.
